# Accuracy evaluation of orthodontic movements with aligners: a prospective observational study

**DOI:** 10.1186/s40510-022-00406-7

**Published:** 2022-04-11

**Authors:** G. Bilello, M. Fazio, E. Amato, L. Crivello, A. Galvano, G. Currò

**Affiliations:** 1 Department of Surgical, Oncological and Oral Sciences, University of Palermo, Palermo, Italy; 2 Palermo, Italy

**Keywords:** Accuracy, Aligner treatment, Orthodontic forces, Orthodontic tooth movement, Orthodontic predictability assessment, Invisalign

## Abstract

**Background:**

Since their introduction in orthodontics, clear aligners have been appreciated by patients, including adults, for their comfort and low aesthetic impact. Despite the enormous mobilization of financial resources all over the world aimed at producing new product lines, few clinical studies or high-quality evidence have been produced regarding the real effectiveness of such treatment. Given the few limited kinds of research on the subject, this study aims to produce and critically evaluate other data, to establish the concrete reliability of clear aligners in orthodontic therapy.

**Results:**

Significant sample sizes were obtained for intrusion, vestibulo/lingual (V/L) crown tipping, and rotation. The overall accuracy for rotation resulted in 86%, ranging from 96% for maxillary central incisors to 70.4% for mandibular first premolars. The intrusion was registered only for anterior teeth; mean predictability was 92%, with the worst result being 86.7% for mandibular canines and the best being 98% for mandibular central incisors. V/L tipping was the most accurate movement: 93.1% of the prescribed movement was completed. Maxillary central incisors showed the lowest accuracy (80.7%), while mandibular central incisors were the highest (97.5%).

**Conclusions:**

The present study provided reassuring data in support of the accuracy of the Invisalign^®^ system. Vestibulo/lingual tipping was the most predictable movement, while rotation of canines, premolars, and lateral incisors were the least predictable. Intrusion resulted highly predictable up to 2 mm. When careful treatment planning follows a correct diagnosis, together with the use of auxiliary features and refinements, the planned results can be achieved in a clinically successful way. Authors believe that there is a major need for greater samples to overcome bias related to variables if we want to answer the unsolved questions, such as the predictability of severe malocclusions treatment.

## Background

Launched on the market in the late 1990s, today clear aligner therapy (CAT) has become a key part of orthodontics thanks to the use of a series of removable, clear thermoplastic appliances to be worn by patients instead of traditional fixed vestibular/lingual braces.

Kesling [1], who had developed a rubber-based tooth positioning appliance in 1946, was the forerunner of this system, until Align Technology (Santa Clara, CA, USA) patented clear aligners in 1998, introducing CAD/CAM technology into orthodontic treatments [2].

ClinCheck is the proprietary software by Align Technology that allows clinicians to plan the treatment digitally and predict the sequence of tooth movement, in terms of time, difficulty, and result. Therefore, through this 3D treatment planning software, the clinician can preview all tooth movements up to the final occlusion as well as showing them to the patient, which can be a great motivation tool.

Since their introduction in orthodontics, clear aligners have been appreciated by patients, including adults, for their comfort and low aesthetic impact. This success has encouraged manufacturers to improve the characteristics of their devices with the introduction of auxiliary features (power ridges, bite ramps, precision cuts, elastics), thus increasing the typologies and severity of malocclusions that can be treated with aligners [3–6]. Today many different cases are considered eligible for orthodontic therapy with these devices: moderate crowding, distalization, resolution of a deep overbite, narrow arches that can be expanded, major rotation, closing/opening of space, etc. [7].

However, despite the enormous mobilization of financial resources all over the world aimed at producing new product lines, few clinical studies or high-quality evidence have been produced regarding the real effectiveness of such treatment.

Among the first papers on Invisalign^®^ (Align Technology), the study by Kravitz et al., evaluating the efficacy of tooth movement with removable polyurethane aligners [8], is one of the most relevant; his group reported a mean accuracy of 41%, the lowest accuracy occurred with extrusion (29.6%) while the highest accuracy occurred with lingual constriction (47.1%), which was the most accurate tooth movement obtained with clear aligners.

Among recent studies, Lombardo et al. [9] report a mean accuracy of movements obtained with F22^®^ clear aligners of 73.6% being the rotation of the mandibular canines the least predictable movement, while the most predictable movements are the mesiodistal tipping of the upper molars and lower premolars.

When the available data are being assessed, clinicians should consider the heterogeneity of the literature in this context, since results refer to several different aligner companies [10].

Given the few limited kinds of research on the subject, this study aims to produce and critically evaluate other data, to establish the concrete reliability of clear aligners in orthodontic therapy.

The main difference between our study and the others available ones is that refinement trays will be included in the assessment, since the authors believe that these corrections are integral part of the treatment.

We set as the null hypothesis that there was no clinical difference between the majority of dental movements planned and the achieved ones.

## Materials and methods

### Trial design, participants, and settings

The present paper is a prospective observational study, approved by the Institutional Review Board of the University of Palermo General Hospital (A.O.U. Policlinico Paolo Giaccone; approval number 2/2020). The study was registered at the German Registry of Clinical Trials (DRKS-ID: DRKS00023865). Informed consent was obtained from all patients.

A total of 10 patients were treated with the same invisible orthodontic system (Invisalign^®^, Align Technology). The subjects selected for the study group were treated in a dental practice in Palermo (Italy).

The sample comprised 215 teeth (105 maxillary and 110 mandibular), which were analysed by overlapping three digital models (pre-treatment, real post-treatment, ideal post-treatment according to setup). The participants included 10 adult patients (3 men and 7 women), whose mean age was 34.8 ± 14.

First of all, a full medical history was documented, followed by a clinical examination. Secondly, diagnostic records—i.e. photographs, orthopantomography, and lateral teleradiographies—were taken to evaluate whether cases were eligible for aligner therapy or not. No fixed appliances or temporary anchorage devices (TADs) were used. Finally, patients deemed acceptable were treated with Invisalign^®^.

The inclusion and exclusion criteria for patient recruitment are reported in Table 1; all subjects were instructed to wear their aligners for 22 h per day and perform the correct oral hygiene procedures. Aligners were replaced every 7 days. In addition to the optimized attachments and interproximal reduction (IPR), auxiliaries (bite ramps, power ridges, elastics) were adopted according to the specific needs of each case (Table 2).

**Table 1:** Criteria for participant’s selection or exclusion

Inclusion criteria	Exclusion criteria
Adult patients	Systemic pathologies
Complete development of every tooth	Active periodontal disease
Complete permanent dentition	Previous orthodontic treatment
No tooth rotations > 30°	Extraction case
No sagittal correction > 4 mm	Structural abnormalities of the craniofacial or dental-alveolar complex
No crowding or diastema > 5 mm	Signs or symptoms of TMJ disorder
Negative pharmacological anamnesis for medications with any effect over bone metabolism	Ongoing pharmacological treatment able to influence orthodontic movement
Negative pathological anamnesis for any illness with effects over the oral cavity	Signs or symptoms of bruxism

**Table 2:** Use of auxiliary features within participants’ treatments

Auxiliary features	Cases
Bite ramps	5/10
Power ridges	10/10
Elastics	4/10
Optimized root control attachments	10/10

When patients completed their first set of aligners, each clinical case was re-evaluated and, if needed, a new ClinCheck simulation was made and a set of “refinement” aligners was used to accomplish all the treatment goals.

Patients needed an average of 25 refinement trays per arch before concluding their orthodontic therapy.

### Digital measurements

**Fig. 1:**
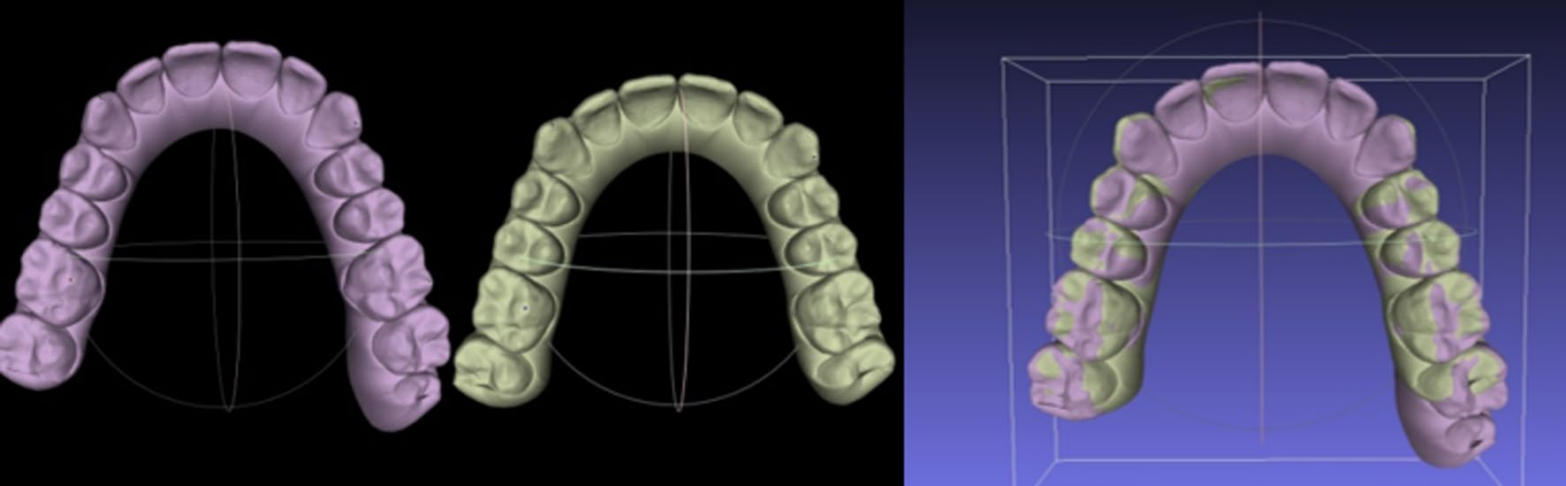
Digital casts superimposition: pre-treatment, real post-treatment and planned post-treatment (Meshlab software)

For each treated dental arch, pre-treatment (T0), post-treatment (T1), and planned post-treatment (Tp) digital casts were available. These 3D models were acquired as STL files via CEREC Omnicam (Dentsply Sirona, Italy) and superimposed by a single operator over stable reference points through Meshlab software (ISTI-CNR, Italy, 2021.05 version) (Fig. 1). Occlusal plane was main reference for superimpositions and vertical displacement measurement, once the models were placed in a 3D Cartesian grid and compared using trigonometry. Correct and repeatable spatial orientation of the models was granted by the use of sagittal and occlusal plane—which do not change after orthodontic treatment—as 3D references. Huanca et al. [11] described how occlusal plane is the best option to achieve a reliable and stable method for superimposition, as palatine folds could serve as reference points but only for the upper arch, while other dental structures could be affected by some movements.

Rhinoceros^®^ software (Robert McNeel & Associates, USA) was used for examining linear and angular measurements of each tooth in a 3D Cartesian grid. Making sections of the superimposed digital casts allowed performing precise measurements.

The different tooth movements examined were: vestibulolingual tipping, mesiodistal tipping, rotation, and vertical displacement (intrusion/extrusion). Despite a major need for information on torque, such dental movement was not examined, as it would have needed a 3D computed tomography (CT) to be properly registered.

**Fig. 2:**
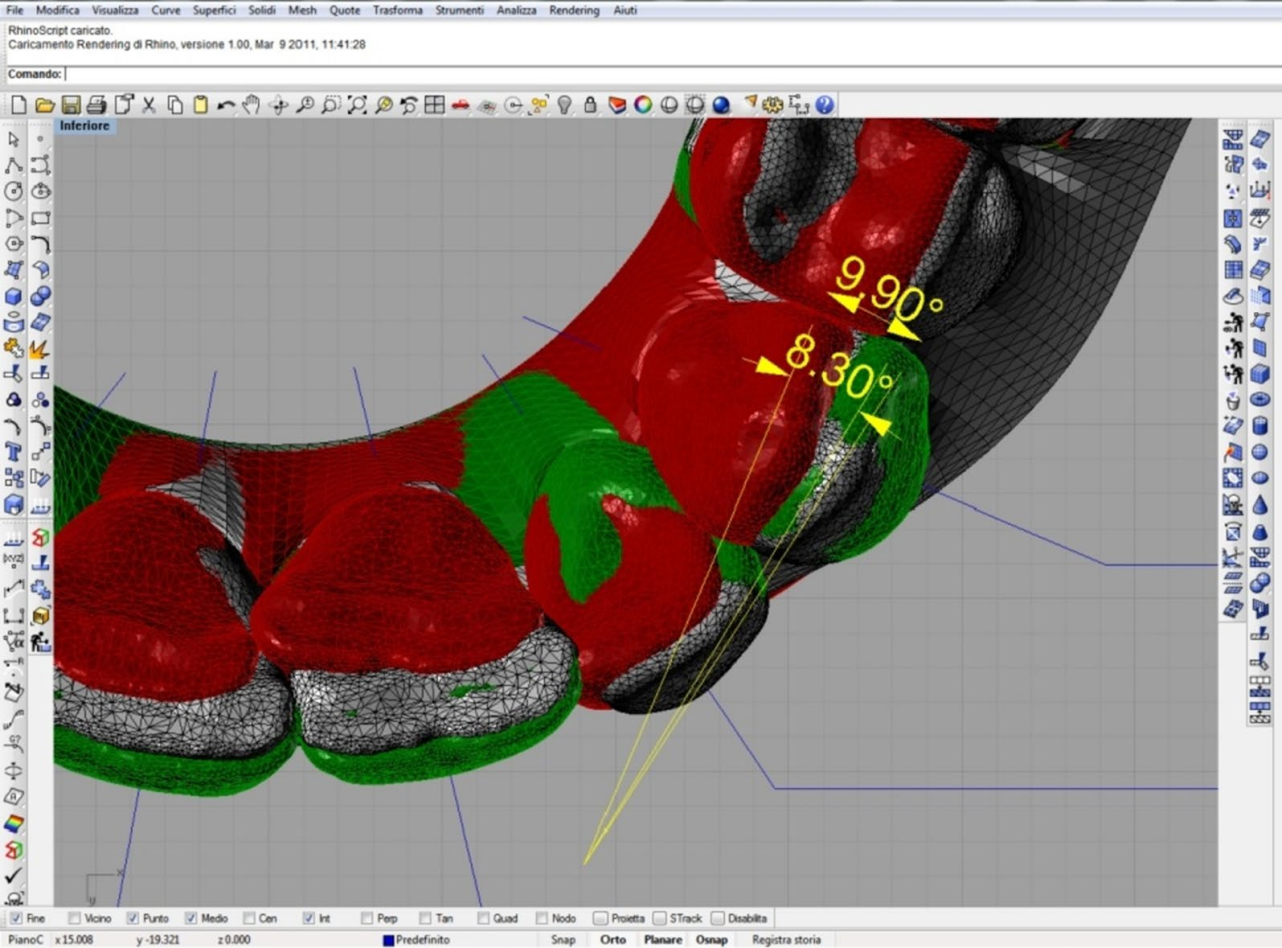
Discrepancy between planned (green) and obtained (white) rotational movement. Colour red identifies the initial position

These superimpositions provided an accurate report on the reliability of the pre-treatment and post-treatment impressions and, above all, the discrepancies between planned treatment and actual results (Fig. 2).

The overall accuracy for every tooth and movement was calculated as follows:

$$\left( {\frac{{Tp\,{\text{position}} - T0 \,{\text{position}}}}{{T1\,{\text{position}} - T0\,{\text{position}}}}} \right) \times 100$$

Whenever accuracy exceeded 100% (as it occurred, for instance, when the vestibularization obtained in the lower-anterior region was greater than the predicted one), the excess was instead subtracted from 100%, obtaining only < 100% values which could be more easily read and interpreted.

**Fig. 3:**
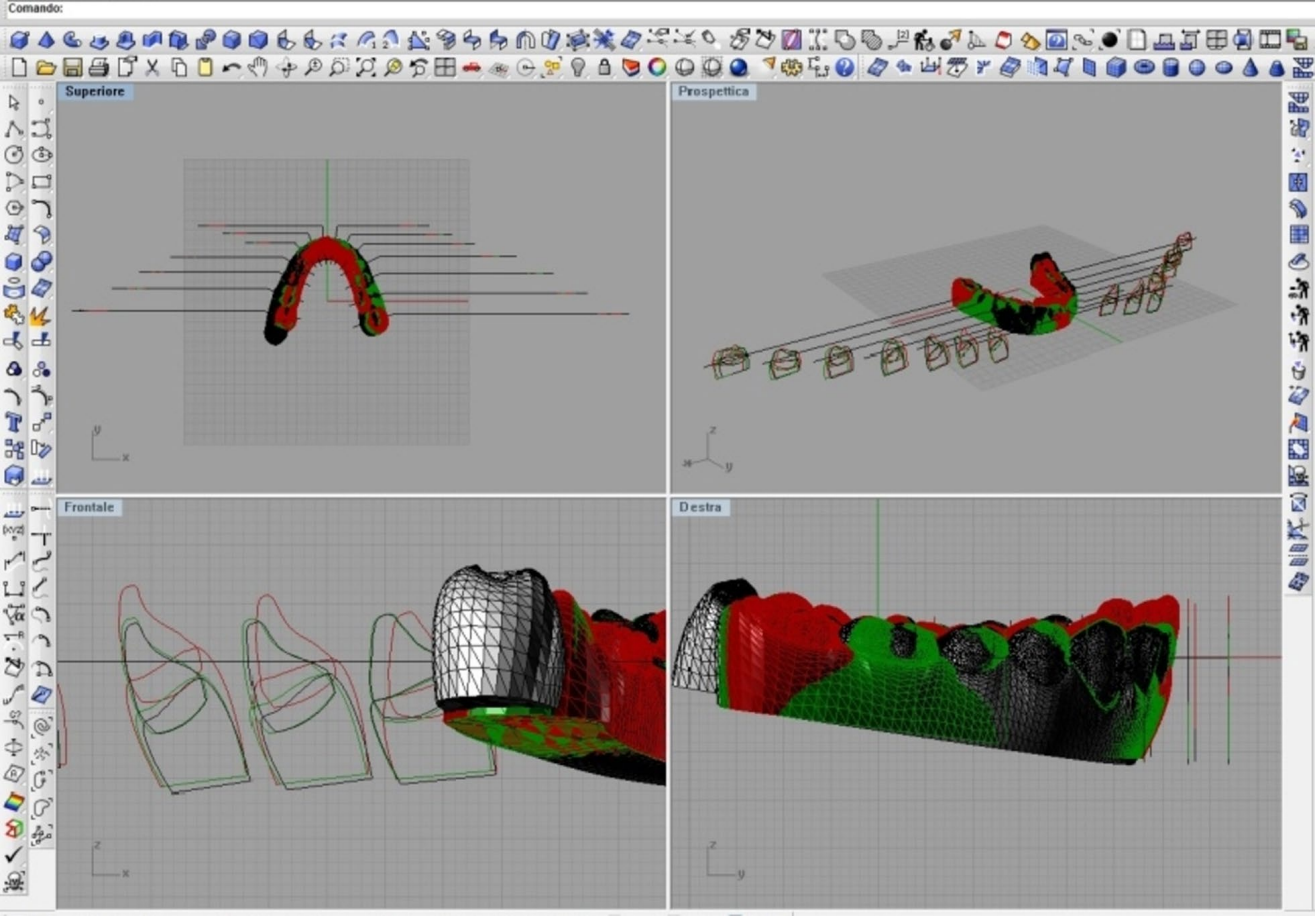
Three models overlapping and sections for every single tooth from an occlusal, perspective, frontal and sagittal point of view with Rhinoceros

Sections were made in a sagittal plane (Fig. 3) using the line passing through the central fossa of molars and premolars, the centre between the mesiodistal margins for the incisors, and the line passing through the cusp for the canines. Tooth inclination was calculated using the facial axis of the clinical crown (FACC) with respect to the occlusal plane.

### Statistical analysis

Statistical analysis was performed using the statistical software package SPSS (SPSS Inc., USA).

Every measurement was repeated by two different trained operators, which allowed assessing that there was no systematic error and the registered values above 0.5 mm and 2° resulted highly reliable and repeatable.

Thus, values below 0.5 mm linear displacement and 2° angular movement were not registered, since they were far from clinical relevance. For every movement and tooth, the Wilcoxon rank-sum test was conducted and *p values* below 0.05 revealed when there was a statistically significant difference between the planned and the actual position of the examined tooth.

## Results

Table 3 shows accuracy percentages sorted by movement and tooth. Authors believed that every tooth should be analysed separately, as it displays unique radicular and crown morphologies as well as supporting alveolar bone histology. These issues must not be underestimated when the response of the teeth to orthodontic forces is being discussed.

**Table 3:** Mean accuracy rate for tooth and movement

Teeth	Rotation %	Intrusion %	V/L tip %
Maxillary central incisors	96.1	91.1	80.7
Maxillary lateral incisors	80.9	91.8	95.9
Maxillary canines	81.0	92.4	92.6
Mandibular central incisors	94.4	98	97.5
Mandibular lateral incisors	90.5	92.0	91.6
Mandibular canines	90.0	86.7	93.8
I maxillary premolars	88.4	(**)	94.4
II maxillary premolars	80.7	(**)	95.1
I maxillary molars	90.6	(**)	90.1
II maxillary molars	92.0	(**)	(**)
I mandibular premolars	70.4	(**)	94.5
II mandibular premolars	82.1	(**)	98.9
I mandibular molars	85.7	(**)	(**)
II mandibular molars	82.2	(**)	(**)

**Sample not suitable for appropriate evaluation

Significant sample sizes were obtained for vertical apical displacement (intrusion), vestibulo/lingual (V/L) crown tipping, and rotation.

When the sample did not result sufficiently represented, the teeth were excluded from the assessment.

Few data were available for both maxillary and mandibular molars, especially the mandibular second molars which were excluded from both intrusion and V/L tip measurements.

The overall accuracy for rotation resulted in 86%, ranging from 96% for the maxillary central incisors to 70.4% for the mandibular first premolars. Nearly half of the examined teeth completed a rotation with over 90% accuracy, while the remaining ones were placed between 80 and 90% (except the mandibular first premolars, with 70.4%).

The intrusion was registered only for anterior teeth, while the sample was not acceptable for posterior teeth. Mean predictability was 92%, with the worst result being 86.7% for the mandibular canines and the best being 98% for the mandibular central incisors.

V/L tipping was the most accurate movement: 93.1% of the prescribed movement was completed. Maxillary central incisors showed the lowest accuracy (80.7%), while mandibular central incisors were the highest (97.5%).

No statistically significant difference was found between planned and actual movement in the majority of cases (Table 4). The entire groups of intrusion and V/L tipping revealed a high precision since the planned and actual final positions resulted statistically homogeneous.

**Table 4:** Statistical relevance of each tooth’s discrepancy between planned and actual position

	*p* value	Significance
*Rotation*		
Maxillary central incisors	0.293	NS
Maxillary lateral incisors	0.04	*
Maxillary Canines	0.018	*
Mandibular central incisors	0.07	NS
Mandibular lateral incisors	0.043	*
Mandibular canines	0.028	*
I maxillary premolars	0.04	*
II maxillary premolars	0.1	NS
I maxillary molars	1	NS
II maxillary molars	0.3	NS
I mandibular premolars	0.03	*
II mandibular premolars	0.1	NS
I mandibular molars	0.2	NS
II mandibular molars	0.3	NS
*Intrusion*		
Maxillary central incisors	0.1	NS
Maxillary lateral incisors	0.32	NS
Maxillary canines	0.2	NS
Mandibular central incisors	0.32	NS
Mandibular lateral incisors	0.11	NS
Mandibular canines	0.06	NS
*V/L TIP*		
Maxillary central incisors	0.063	NS
Maxillary lateral incisors	0.345	NS
Maxillary canines	0.11	NS
Mandibular central incisors	0.075	NS
Mandibular lateral incisors	0.12	NS
Mandibular canines	0.5	NS
I maxillary premolars	0.3	NS
II maxillary premolars	0.1	NS
I maxillary molars	0.9	NS
I mandibular premolars	0.5	NS
II mandibular premolars	0.4	NS

The asterisk indicates those values statistically significant (*p* < 0.05)

Nevertheless, some final positions were found to be significantly different from the predicted ones. In particular, *p values* < 0.05 were calculated for the intrusion of some teeth: maxillary lateral incisors, maxillary canines, mandibular lateral incisors, mandibular canines, maxillary first premolars, and mandibular first premolars.

## Discussion

The reliability of 3D digital planning has always been the centre of the debate over aligner therapy, since its introduction. At an early stage, in 2005, Djeu et al. [12] highlighted a clear difference between Invisalign^®^ system and multibracket treatment, with the latter being significantly more effective in achieving good occlusal relationships and sagittal discrepancies. Recent systematic reviews, for instance, the 2019 study by Robertson et al. [13], found that clear aligners are as effective in the resolution of mild to moderate malocclusions as fixed orthodontics, although braces might be the best choice for complex malocclusions.

Different results throughout the years reflect the significant improvements in technology, software, and materials, not to mention the fact that new knowledge and experience allow clinicians to properly choose among aligners and other orthodontic tools.

The present study aims to give a reliable evaluation of how accurate Invisalign^®^ can be in obtaining the prescribed movements. Appropriate sample size was obtained for rotation, intrusion of anterior teeth, and V/L tipping. Clinical experience and literature evaluation allow authors to maintain that these are the most prescribed movements when aligners are properly used. Major posterior intrusion, mesiodistal (M/D) translation, or torque movement are today considered contraindications [14] for the use of aligners, but the accuracy of the other movements is far from being clear.

Our results suggest that all examined movements can be achieved with high-rate precision, especially the intrusion of anterior teeth and V/L tipping, which showed an overall completion rate being, respectively, 92% and 93.1%, compared to prescription. Significantly lower results were obtained for rotation (86%) with the lowest accuracy regarding rotation of mandibular first premolars (70.4%).

Despite the great interest in the matter, little evidence has been produced regarding the exact amount of movements we can obtain from clear aligners with respect to the ones indicated in the 3D initial project.

Tepedino et al. [15], using Nuvola^®^ system, put the focus on the importance of re-evaluation of results every 12 steps before proceeding to the next set of aligners. Among clinical papers, the study by Lombardo et al. [9] presents the most remarkable protocol and results: they obtained a mean accuracy of 73.6%. The least predictable movement was lower canine rotation, while the most predictable resulted to be the M/D tipping of lower premolars.

In 2015, Rossini et al. [16] found reduced predictability for rotations, especially for rounded teeth such as canine and premolars, and extrusion, while intrusion and distalization up to 1.5 mm proved to be more accurate.

What results from the most recent systematic reviews of the efficacy of tooth movement with Invisalign^®^, among which the prospective follow-up study by Haouil et al. [17], is that maximum efficiency is obtained in V/L tipping, while the lowest involves the rotation of the canines and premolars. However, though these findings are better than the data obtained in the previous part of this study [3], they do not change what we know about the most and least effective movements.

To our knowledge, our results may overcome the data already available in the literature, despite the fact strengths and weaknesses of clear aligners proved to be the same. Authors believe that this is due to the use of refinements, i.e. new sets of aligners worn by the patients when the first sequence did not lead to satisfying results. Refinements are the counterpart of finishing procedures in fixed appliances; several methods of completing a multibracket treatment are used [18], but the need for finishing in most cases is undisputed. Therefore, since refinements are common events during orthodontic treatment with Invisalign^®^, they should be included in the evaluation of accuracy as done in this study.

Furthermore, some auxiliary features helped in achieving prefigured treatments goals.

The majority of intrusions did not exceed 2 mm, which can explain, together with the frequent use of bite ramps and pressure areas, the extremely high success rates.

Since aligners biomechanics is based on the pressure generated by the deformation of the aligner’s surface over the crown of the tooth, it seems logical that V/L tipping was shown as the most accurate movement in most papers.

With regard to rotation, the recent adoption of optimized rotation attachments aimed to create a couple of forces and the momentum that the tooth needs to make a pure rotation on its axis. However, this strategy was not sufficient, as incomplete rotations were the main reason for the need for refinements in our sample. In these cases, the clinician found it useful to perform a slight IPR between canines and the adjacent teeth.

A key fact when interpreting these data is that no movement in orthodontics is pure, but each one consists of several components; when evaluating intrusion, if the tooth simultaneously goes through a vestibularization—implying a “relative” intrusion—the total amount of intrusion will be the sum of the two movements, and so on. Therefore, every result that isolates a single movement should be judged as a simplification of a complex reality, considering that studying the overall movement—as it should be made—would not lead to an advancement in our knowledge about what can and what cannot be predictably done through aligners.

The main limitation to this study is the reduced sample that did not allow the evaluation of major corrections, especially in posterior teeth that are infrequently involved in great movements. Nevertheless, many hybrid approaches are being tested to support posterior corrections in association with clear aligners; therefore, future papers are likely to bring innovations to this field.

A further limitation is the risk of overestimation of Invisalign^®^ efficacy. The fact that no limits were put to the number of refinements used implied that from an average of 21,2 aligners per arch, the final mean number of steps per arch increased to 50. This meant that the planned movements were achieved, but at a higher cost of time than the initial evaluation. Nonetheless, the authors want to stress how this fact did not lead to periodontal problems, root resorption, or further economic costs, in contrast to what can occur with fixed appliances.

A last significant limitation is a knowledge that ClinCheck^®^ cannot be used as a universal predictor of results. In fact, on the one hand, the huge amount of variables, in the response of the tissues to orthodontic forces, makes it impossible to predict an exact position; on the other hand, a 3D simulation can be used in combination with clinical expertise to set an initial goal as a starting point for the evaluation of the individual responses.

## Conclusions

The present study provided reassuring data in support of the accuracy of the Invisalign^®^ system; when careful treatment planning follows a correct diagnosis, together with the use of auxiliary features and refinements, the planned results can be achieved in a clinically successful way.

The data extrapolated from this analysis, however, agree with other studies, revealing a discrepancy between the planned movements and the results obtained.

- Vestibulolingual tipping was the most predictable movement, reaching an accuracy of 95.9% for the lower arch and 94.6% for the upper arch. Intrusions reached a mean accuracy of 92.2% and 94.3% in the lower and upper arch, respectively. Rotations achieved a mean accuracy of 86.0%.
- The least predictable movement was the rotation of the premolars, canines, and lateral incisors: the upper canine had a mean accuracy of 81.0%, the lateral incisor 80.9%, the second upper premolar 80.7%, the lower first premolar 70.4%.
- There was no statistical significance among tooth movements, except for the rotation of upper and lower lateral incisors, canines, and premolars.
- The intrusion was highly predictable up to 2 mm, above which the predictability of the movement was reduced.

In conclusion, the results obtained from this clinical study considerably contribute to the ongoing debate. It is desirable that in the future greater samples will be analysed to overcome bias related to variables (inter-and intra-arch differences, "relative" movements, radicular movement control), thus obtaining more reliable answers to the clinical questions that remain unsolved to date, the majority of which are related to the treatment of severe malocclusions.

## Data Availability

The data set supporting the conclusions of this article is included within the article. Further data sets are available from the corresponding author on reasonable request.
